# Corticosterone and decision-making in male Wistar rats: the effect of corticosterone application in the infralimbic and orbitofrontal cortex

**DOI:** 10.3389/fnbeh.2014.00127

**Published:** 2014-04-21

**Authors:** Susanne Koot, Magdalini Koukou, Annemarie Baars, Peter Hesseling, José van ’t Klooster, Marian Joëls, Ruud van den Bos

**Affiliations:** ^1^Brain Center Rudolf Magnus, Department of Neuroscience and Pharmacology, University Medical Centre UtrechtUtrecht, Netherlands; ^2^Division Behavioural Neuroscience, Department of Animals in Science and Society, Faculty of Veterinary Medicine, Utrecht UniversityUtrecht, Netherlands; ^3^Department of Organismal Animal Physiology, Radboud University NijmegenNijmegen, Netherlands

**Keywords:** decision-making, Iowa Gambling Task, corticosterone, infralimbic/orbitofrontal cortex, rat

## Abstract

Corticosteroid hormones, released after stress, are known to influence neuronal activity and produce a wide range of effects upon the brain. They affect cognitive tasks including decision-making. Recently it was shown that systemic injections of corticosterone (CORT) disrupt reward-based decision-making in rats when tested in a rat model of the Iowa Gambling Task (rIGT), i.e., rats do not learn across trial blocks to avoid the long-term disadvantageous option. This effect was associated with a change in neuronal activity in prefrontal brain areas, i.e., the infralimbic (IL), lateral orbitofrontal (lOFC) and insular cortex, as assessed by changes in c-Fos expression. Here, we studied whether injections of CORT directly into the IL and lOFC lead to similar changes in decision-making. As in our earlier study, CORT was injected during the final 3 days of the behavioral paradigm, 25 min prior to behavioral testing. Infusions of vehicle into the IL led to a decreased number of visits to the disadvantageous arm across trial blocks, while infusion with CORT did not. Infusions into the lOFC did not lead to differences in the number of visits to the disadvantageous arm between vehicle treated and CORT treated rats. However, compared to vehicle treated rats of the IL group, performance of vehicle treated rats of the lOFC group was impaired, possibly due to cannulation/infusion-related damage of the lOFC affecting decision-making. Overall, these results show that infusions with CORT into the IL are sufficient to disrupt decision-making performance, pointing to a critical role of the IL in corticosteroid effects on reward-based decision-making. The data do not directly support that the same holds true for infusions into the lOFC.

## Introduction

Stress is the subjective experience of actual or potential, physical or psychological threat. Upon stress, a physiological response is triggered which prepares the organism to deal with the changing environment (Joëls and Baram, 2009; Joëls et al., 2011). As part of the stress response, the adrenal cortex secretes glucocorticosteroid hormones in response to adrenocorticotropic hormone from the pituitary gland. Neurons may therefore receive high levels of corticosteroids (cortisol in humans, corticosterone (CORT) in most rodents), which influence neuronal activity and produce a wide range of effects upon the brain (McEwen, 2006; Groeneweg et al., 2011). Altered gene transcription mediates many of the glucocorticoid actions, but a range of effects on behavior and endocrine output occurs within minutes (Groeneweg et al., 2011). Ultimately, glucocorticosteroids restore homeostasis in the aftermath of stress by diverting energy supply to challenged tissues and control the excitability of neuronal networks (de Kloet et al., 1999).

Cognitive processes, like memory (Joëls et al., 2006; Schwabe et al., 2012) and decision-making (Starcke and Brand, 2012) have been shown to be affected by stress. Effective decision-making requires the cooperation of several cognitive skills such as planning, initiating and monitoring goal-directed behavior (Rivalan et al., 2011). One way to study decision-making performance in humans is through the Iowa Gambling Task (IGT; Bechara et al., 1994). Due to combining the uncertainty of outcome with a conflict between short-term and long-term benefit, this task is widely used as a simulation of real-life decision-making (Brand et al., 2007). Stress has been repeatedly linked to suboptimal decision-making. Men have been shown to display more risk-taking behavior under acutely stressful conditions in both the IGT (Preston et al., 2007; van den Bos et al., 2009) and other decision-making tasks (Balloon Analogue Risk Task, Lighthall et al., 2009; Game of Dice Task, Starcke et al., 2008; Starcke and Brand, 2012). Specifically, cortisol reactivity correlated negatively with decision-making performance in the IGT in men, i.e., the higher the salivary cortisol levels, the poorer task performance (van den Bos et al., 2009; conform Pabst et al., 2013; van den Bos et al., 2014). These effects may be mediated by changes in activity in prefrontal areas (Arnsten, 2009; Joëls et al., 2012).

In order to more precisely examine the effect of corticosteroids on decision-making, and particularly the relevance of the time at which hormone levels were raised relative to behavioral testing, we tested the effect of CORT treatment in a rodent version of the IGT (rIGT; Koot et al., 2013). As we expected that CORT would exert its effect on decision-making through actions in the medial prefrontal cortex (mPFC; see Koot et al., 2013 for rationale) and the mPFC has been indicated to be involved in task-performance in the second half of the task (de Visser et al., 2011a,b; van Hasselt et al., 2012), we injected CORT in the second half of the task. We found impaired decision-making of male rats tested 30 min after systemic injections of CORT (1 mg/kg subcutaneously): unlike vehicle treated rats, CORT treated rats did not decrease their number of visits to the disadvantageous arm across trial blocks (Koot et al., 2013). CORT administration did not affect choices for empty arms, which indicates that only reward-related decision-making was affected by CORT. No effects were found when CORT was administered 180 min prior to testing, so only rapid corticosteroid actions impaired decision-making. This effect of CORT injections on decision-making was accompanied by a significant increase in c-Fos expression in the lateral orbitofrontal cortex (lOFC) and insular cortex and a strong trend for an increase in the infralimbic cortex (IL; ventral region of the mPFC); CORT treatment in itself did not change c-Fos expression. In other words, only when animals were exposed to the rIGT, CORT injections were associated with an increase in neuronal activity in the lOFC, insular cortex and IL; no such effect was found in a group of home cage control animals which received CORT injections but were not tested in the rIGT.

As follow up of this study, here we applied CORT locally into the IL and lOFC to assess the effects of CORT on these areas directly. We focused on these two areas, as pilot experiments showed that we were not able to reliably target the subregion of the insular cortex. Similar to the design of our previous study (Koot et al., 2013), we infused CORT during the second half of the rIGT and administered the drug bilaterally into either the IL or the lOFC. We reasoned that if the effects of systemic CORT injections on decision-making result from direct effects of CORT in these areas, local CORT infusions would also result in disrupted reward-related decision-making performance, i.e., a failure to decrease the number of choices for the disadvantageous arm and no effects on empty arm choices.

## Materials and methods

### Animals

Male Wistar WU rats (*n* = 84; Charles River, Sulzfeld, Germany; 9 weeks of age) were housed individually in Macrolon type IV cages with sawdust bedding under a reversed 12 h light/dark cycle (lights off at 07:00 h) under controlled conditions (temperature 21 ± 2°C, relative humidity 60 ± 15%). Paper tissues and a shelter were provided as cage enrichment. Food and water were available ad libitum, except during testing (see below). A radio provided background noise. Behavioral testing started at least 1 h after dark onset. All experiments were approved by the Animal Ethics Committee of Utrecht University and were conducted in agreement with Dutch laws (Wet op de Dierproeven, 1996) and European regulations (Guideline 86/609/EEC).

### Surgery

After 2.5 weeks of habituation to the facility and human handling, surgery took place. Rats were anaesthetised under red-light conditions using a mixture of ketamine (60 mg/kg, i.p., Narketan, Vétoquinol S.A., France, 100 mg/ml ketamine) and dexmedetomidine (0.15 mg/kg, i.p., Dexdomitor, Pfizer Animal Health BV, Capelle a/d IJssel, The Netherlands, 0.5 mg/ml dexmedetomidine hydrochloride), including buprenorphine (0.05 mg/kg, i.p., Temgesic, RB Pharmaceuticals Limited, 0.3 mg/ml buprenorphine) as additional analgesia. As soon as the pedal reflex was absent, the rat was transported to the surgery room and, after intubation, anaesthesia was maintained with isoflurane in 100% O_2_. The animals received 8 ml of saline (s.c.) to support normal fluid balance, and eye ointment (Ophtosan Oogzalf, Produlab Pharma Raamsdonkveer, ASTfarma BV, Oudewater, The Netherlands; 10,000 IE of vitamin A palmitate per gram). For surgery, the animal was positioned in the stereotactic apparatus (model 963, Ultra Precise Small Animal Stereotaxic, David Kopf Instruments, Tujunga, CA, USA). Body temperature was monitored using a rectal probe thermometer and maintained at 37–38°C with an adjustable electrically heated mattress. Respiratory rate, and inspired and expired CO_2_ were monitored continuously and anaesthetic administration was adjusted appropriately. Rats were implanted bilaterally with stainless steel guide cannulas (length: 5 mm; 22 ga; Plastics One type C313GRL, Plastics One Inc., Roanoke, VA, USA) aimed at the IL or lOFC. The IL was targeted under a lateral angle of 30° using the following coordinates: anteroposterior (AP): +3.2 mm relative to Bregma; mediolateral (ML): ±2.8 mm; dorsoventral (DV): 4.4 mm below skull surface. The coordinates used for the lOFC were: AP: +4.3 mm; ML ±2.7 mm; DV: −3.6 mm; no angle. In both cases, the nose was set at −3.3 mm. All coordinates were adapted from the atlas of Paxinos and Watson (2005) to our rats.

After surgery, anaesthesia was antagonized with atipamezole (0.6 mg/kg, i.p., Antisedan, Pfizer Animal Health BV, Capelle a/d IJssel, The Netherlands, 5 mg/ml atipamezole hydrochloride). Rats were returned to their home cages once they regained locomotion. Postoperative analgesia consisted of meloxicam (0.2 mg/kg, s.c., Metacam, Boehringer Ingelheim, Alkmaar, The Netherlands, 5 mg/ml meloxicam) at 24 h intervals for 2 days after surgery, plus buprenorphine (0.05 mg/kg, s.c.) at 12 h intervals for 3 days after surgery. All rats were allowed to recover for 2 weeks before behavioral testing started.

### Behavioral procedures

Experiments were run as described previously (de Visser et al., 2011a,b; Koot et al., 2013) in order to keep the procedure similar to earlier used protocols. Therefore, after a period of 2 weeks, rats were first tested on the elevated plus maze (EPM) and subsequently in the rodent version of the IGT (rIGT). Because anxiety, as measured by EPM performance, was earlier found to correlate with rIGT behavior (Rivalan et al., 2009; de Visser et al., 2011a), we compared anxiety levels between batches of rats to promote that they were comparable prior to saline or corticosteroid treatment. We refer to de Visser et al. (2011a) for detailed descriptions of the EPM protocol and analyses. We analysed per cent time spent on the open arm as a measure of anxiety (de Visser et al., 2011a). No significant differences were found between batches (data not shown); thus all batches were included for further analysis.

Rats were tested in the rIGT 1 week after EPM exposure (starting on Mondays; see de Visser et al., 2011a). We allowed a brief recovery time after the EPM to avoid possible interference of this test with subsequent testing in the rIGT. Since the exposure to the EPM was only brief, we assume that a 1 week interval was sufficiently long. The rIGT apparatus, made of gray polyvinyl chloride, consisted of a start box, choice area and four arms. Before testing, rats were habituated to the apparatus in a 10 min free exploration trial. Two days later they were mildly food deprived (90–95% of free feeding body weight) and tested for 9 days. Testing took place between 9:00–15:00 h (lights off at 7:00 h). Testing did not occur during weekend days. Food was freely available on these days, but rats were returned to their restricted diet the day before testing continued.

A trial started by lifting the slide door of the start box. Rats could freely enter the choice area of the apparatus and choose one of the four arms. To help rats differentiating arms, distinct visual cues (10 × 10 cm; cross or circle in black or white) were placed to the side of the wall at the entrance of the arms at a height of 15–20 cm from the floor (see Figure 2 in van den Bos et al., 2006). When rats had entered a choice arm with their full body, including their tail, the arm was closed. At the end of the arm, rats could obtain pellets (baited arms) or nothing at all (empty arms). Each trial lasted maximum 6 min (inter-trial interval: 30 s). Rats received a total of 120 trials: 10 trials on days 1–6 and 20 trials on days 7–9. By the time rats reached the second half of the task, i.e., days 7–9, a session lasted 12 min at most. The number of trials per session was increased to 20 trials per session in the second half of the task, as by that time rats had become faster in their choices, allowing a higher number of trials per session (conform Koot et al., 2013).

Rewards were 45 mg sugar pellets (F0042, Bio-serv Inc, Frenchtown, NJ, USA); punishments were quinine-treated sugar pellets that were unpalatable but not uneatable. Rats were habituated to the sugar pellets in the week prior to the first rIGT session in their home cage daily, followed by a single session of providing two sugar pellets in a novel empty Macrolon type-III cage, which all rats did eat. During rIGT testing most rats consumed the quinine-treated sugar pellets once, but left them uneaten after tasting them briefly. Typically, rats that consistently eat quinine-treated sugar pellets are removed from statistical analyses; however, no rats engaged in this behavior in the current study.

Of the four arms in the maze, two arms were baited and two arms were empty. The two empty arms were included to measure non-reward related exploration (de Visser et al., 2011a,b,c). The two baited arms consisted of a long-term disadvantageous arm and a long-term advantageous arm. In the disadvantageous arm, rats received occasional big rewards (3 sugar pellets in 1 out of 10 trials) among frequent punishments (3 quinine-treated sugar pellets in 9 out of 10 trials). In the advantageous arm, rats received frequent small rewards (1 sugar pellet in 8 out of 10 trials) and infrequent punishments (1 quinine-treated sugar pellet in 2 out of 10 trials). The positions of the baited and empty arms, as well as the advantageous and disadvantageous arm were counterbalanced across rats.

Performance parameters were: (1) the number of visits to the empty arms as a fraction of the total number of trials per block of 20 trials and (2) the number of visits to the disadvantageous arm as a fraction of the total visits to the baited arms per block of 20 trials. To unravel the potential mechanisms underlying behavioral effects of CORT injections, we measured some additional parameters. First, the total number of switches between different arms per block of 20 trials was calculated as measure of exploratory behavior (de Visser et al., 2011a). Second, responses to encounters with sugar pellets or quinine-treated sugar pellets in the advantageous and disadvantageous arm were measured as win-stay/lose-shift behavior per block of 20 trials (de Visser et al., 2011a). Blocks of 20 trials were chosen to obtain a sufficient number of encounters with sugar and quinine-treated sugar pellets. Thus, when rats encountered a sugar reward, their subsequent choice was scored as a win-stay when they revisited the advantageous arm. When rats encountered a quinine punishment, their subsequent choice was scored as a lose-shift when they switched to another arm. Win-stay and lose-shift behavior was calculated as a fraction of the number of encounters with either sugar (win) or quinine (loss). Thus, values of 1 indicate win-stay and lose-shift tendencies only, while values of 0 indicate win-shift and lose-stay tendencies only.

### Intracerebral infusions

On the last 3 days of the rIGT, i.e., the second half of the rIGT, rats received intracerebral infusions of CORT or vehicle. As the present study is a follow up of our earlier study, where we injected CORT or vehicle (systemically) in the second half of the task, we here infused CORT or vehicle in these sessions as well, i.e., the final three trial blocks of 20 trials each (Koot et al., 2013). Rats were infused bilaterally with a solution of CORT (5 ng/μL; Sigma-Aldrich) or vehicle solution in a volume of 0.5 μL per hemisphere, approximately 25 min before testing. The dose of 5 ng/μL CORT was chosen as in previous infusion studies this dose resulted in significant effects compared to other doses, i.e., 5 ng/μL as opposed to 2 or 10 ng/μL resulted in enhanced memory when infused in the insular cortex, basolateral amygdala or dorsal striatum (Miranda et al., 2008; Quirarte et al., 2009). CORT was first dissolved in ethanol and then diluted in saline to reach the intended concentration. The final concentration of ethanol was 2%. The vehicle solution consisted of 2% ethanol in saline. The infusions targeted either the IL or the lOFC. Hamilton 10-μL syringes were used, attached to tubing and an injection needle of 6 mm long, thus protruding 1 mm beyond the tip of the guide cannulas. Before the infusions, longer injection needles (7 mm) were inserted in the cannulas to prevent blockage. Then, a mechanical pump (Harvard Apparatus, serial number: B-17020) was used to perform the infusions. A volume of 0.5 μL per hemisphere was infused at a rate of 0.4 μL/min. The needles were left in place for 60 s in addition to allow for diffusion. During the whole procedure, the rats were held by the experimenter and slightly restrained to prevent abrupt movements. After infusion rats were returned to their home cage, before they were tested in the rIGT.

### Histology

After completion of the experimental protocol, rats were decapitated and brains were quickly frozen in liquid (−80^°^C) 2-methylbutane and stored at −80^°^C. Coronal sections (20 μm) were cut on a cryostat (Leica CM 3050S), mounted on Menzel SuperFrost Plus slides (MenzelGmbH&Co, Braunschweig, Germany) and stained with cresyl violet. Injection sites were verified with reference to the neuro-anatomical atlas of Paxinos and Watson (2005).

### Statistical analyses

IBM SPSS Statistics 20 for Windows was used for the data analysis. Specific tests are indicated in the Results section. Whenever sphericity was violated, degrees of freedom were adjusted. Significance for all tests was set at *p* ≤ 0.05 (two-tailed), except for the one-sample *t*-tests (Bonferroni corrected *p* ≤ 0.017 (two-tailed)). Results are expressed as mean ± SEM.

## Results

### General

**Figure 1:**
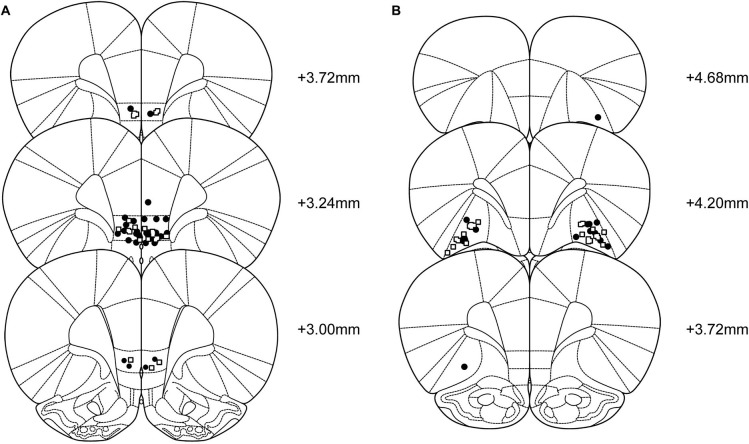
**Representation of the location of the infusions into the (A) infralimbic cortex (CORT: black circles, *n* = 21; vehicle: open squares, *n* = 13), and (B) lateral orbitofrontal cortex groups (CORT: black circles, *n* = 11; vehicle: open squares, n=12).** Sections correspond to the atlas of Paxinos and Watson (2005).

Forty-eight rats were cannulated targeting the IL and 36 targeting the lOFC. Six rats died during surgery or recovery (IL *n* = 2, lOFC *n* = 4). In the IL-infused group, 12 additional rats were excluded; 5 due to incorrect placement of the cannulas, 1 due to loss of 1 guide cannula, 1 because the target region of the cannulas was impossible to verify, 2 due to large infections in the brain, 2 due to not completing the rIGT and 1 due to health problems affecting the performance. This resulted in *n* = 34 rats used for the analysis (CORT = 21, vehicle = 13). In the lOFC-infused group, 9 additional rats were excluded; 5 due to incorrect placement of the cannulas, 1 due to difficulties verifying the location of the cannulas and 3 due to not completing the rIGT. This resulted in *n* = 23 rats used for the analysis (CORT = 11, vehicle = 12). Figure 1 shows the location of injection needle tips in the IL and lOFC of rats tested in the rIGT and included in the analyses.

### Rat Iowa Gambling Task (rIGT)

**Figure 2:**
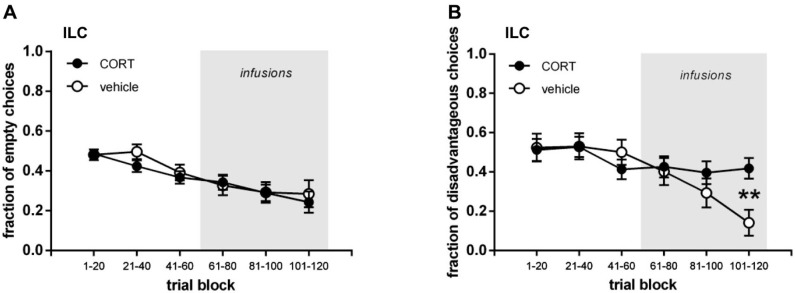
**Infralimbic cortex (IL) cannulated groups. (A)** Mean (±SEM) fraction of visits to empty arms. **(B)** Mean (±SEM) fraction of visits to disadvantageous arm. ** *p*=0.001.

**Figure 3:**
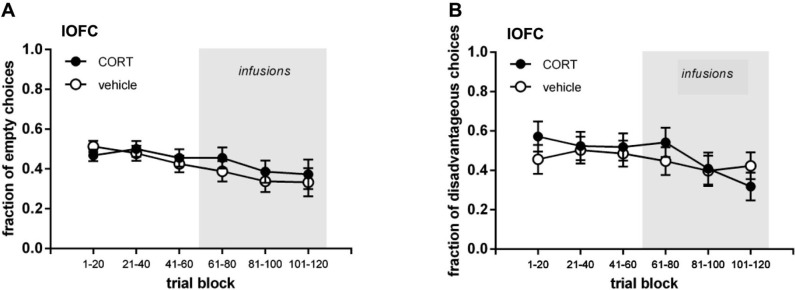
**Lateral orbitofrontal cortex (lOFC) cannulated groups. (A)** Mean (±SEM) fraction of visits to empty arms. **(B)** Mean (±SEM) fraction of visits to disadvantageous arm.

Figures 2 and 3 show the performance of rats infused with CORT or vehicle in the IL and lOFC. We depicted their performance in the first three trial blocks (t1–60) and in the last three trial blocks (t61–120), both for visits to the empty arms and disadvantageous arm.

#### Baseline (trials 1–60)

To assess whether animals demonstrated systematic task-learning in the first half of the task, the fraction of visits to the empty arms and the fraction of visits to the disadvantageous arm per trial block were statistically tested against chance level (fraction = 0.5; one-sample *t*-test; Bonferroni corrected *p* ≤ 0.017). Rats intended to be injected with CORT in the IL demonstrated task-learning for the empty arms in trial blocks 21–40 (*p* = 0.012) and 41–60 (*p* < 0.001). No other significant differences in either vehicle or lOFC (CORT/vehicle) intended rats were observed (all *p* ≥ 0.031).

To check whether any baseline differences between treatment groups prior to the infusions existed, a three-way repeated measures ANOVA was run for the fractions of visits to empty arms and fraction of visits to the disadvantageous arm for all animals on the first three trial blocks. Trial block was set as within-subjects factor, brain region (IL, lOFC) and treatment (CORT, vehicle) as between-subjects factors. No differences in performance existed between the IL and lOFC-cannulated groups or between the intended treatment groups before treatment started, and all rats visited the empty arms less often across trial blocks (empty arms: trial block *p* = 0.001, all other *p* ≥ 0.198; disadvantageous arm all *p* ≥ 0.267). A two-way ANOVA on the last trial block before treatment (t41–60) with brain region and treatment as between-subjects factors confirmed this, as no significant differences between the above-mentioned groups were found (all main effects and interactions: *p* ≥ 0.136).

#### Treatment (trials 61–120)

To assess whether animals demonstrated systematic task-learning in the second half of the task, the fraction of visits to the empty arms and the fraction of visits to the disadvantageous arm per trial block were statistically tested against chance level (fraction = 0.5; one-sample *t*-test; Bonferroni corrected *p* ≤ 0.017). In the IL infused groups both the vehicle and CORT infused rats demonstrated task-learning in all three trial blocks for the fraction of visits to the empty arms (all *p* ≤ 0.017). For the fraction of visits to the disadvantageous arm the vehicle infused rats demonstrated task-learning in trial blocks 81–100 (*p* = 0.015) and 101–120 (*p* < 0.001), while CORT infused rats did not (all *p* ≥ 0.122). In the lOFC infused groups, for the fraction of visits to the empty arms vehicle infused rats showed task-learning in all three trial blocks (all *p* ≤ 0.015), while the CORT infused rats only did so in trial block 81–100 (*p* = 0.007). For the fraction of visits to the disadvantageous arm neither vehicle infused nor CORT infused rats demonstrated task-learning in the different trial blocks (all *p* ≥ 0.040).

In the second half of the task, regardless of treatment, all rats decreased their fraction of visits to the empty arms, with IL-cannulated rats tending to visit the empty arms less often than lOFC-cannulated rats (Figures 2A and 3A; three-way repeated measures ANOVA (brain region, treatment; repeated: trial block; Huynh-Feldt adjusted): trial block *F*_(1.89,   100.18)_ = 3.407, *p* = 0.040; brain region *F*_(1,53)_ = 3.198, *p* = 0.079; all other interactions and main effects: *p* ≥ 0.509). When the IL and lOFC infused groups were analyzed separately with two-way repeated measures ANOVAs, no differences between CORT and vehicle treatment appeared either (IL: all interactions and main effects *p* ≥ 0.164; OFC: all interactions and main effects *p* ≥ 0.189). Thus, CORT had no effect on empty arm choices either infused in the IL or lOFC.

In contrast, Figures 2B and 3B show that rats infused with vehicle in the IL visited the disadvantageous arm less often over trial blocks in contrast to the rats infused with CORT in the IL, while rats infused with vehicle and CORT in the lOFC only slightly decreased their visits to the disadvantageous arm with no differences between groups. This was confirmed by a three-way interaction term (three-way repeated measures ANOVA (brain region, treatment; repeated: trial block): trial block ^*^ treatment ^*^ brain region: *F*_(2,106)_ = 5.836, *p* = 0.004; trial block: *F*_(2,106)_ = 7.567, *p* = 0.001; all other interactions and main effects *p* ≥ 0.181). Moreover, this was confirmed when IL and lOFC infused groups were analyzed separately with two-way ANOVAs (IL infused group: trial block: *F*_(2,64)_ = 4.461, *p* = 0.015; trial block ^*^ treatment: *F*_(2,64)_ = 4.107, *p* = 0.021; treatment: *F*_(1,32)_ = 2.930, *p* = 0.097; lOFC infused group: trial block: *F*_(2,42)_ = 3.590, *p* = 0.036; trial block ^*^ treatment: *F*_(2,42)_ = 2.198, *p* = 0.124; treatment: *F*_(1,21)_ = 0.000, *p* = 0.999).

In an attempt to delineate the mechanisms underlying the significant difference between CORT and vehicle infused IL rats, we analyzed switches and win-stay and lose-shift behavior in trial block 101–120 in the IL infused group. No differences were present between CORT infused rats and vehicle infused rats with respect to switches (CORT 9.10 ± 0.94 vs. vehicle 8.77 ± 1.19; one-way ANOVA, treatment *F*_(1,33)_ = 0.046, *p* = 0.831). Similarly, no differences were found between CORT infused rats and vehicle infused rats with respect to lose-shift (CORT 0.65 ± 0.07 vs. vehicle 0.81 ± 0.12; one-way ANOVA, treatment *F*_(1,25)_ = 1.176, *p* = 0.289) or win-stay behavior (CORT 0.50 ± 0.06 vs. vehicle 0.65 ± 0.07; one-way ANOVA, treatment *F*_(1,26)_ = 2.303, *p* = 0.142).

Visual inspection of the dataset suggested that vehicle treated rats within the lOFC infused group did not decrease their visits to the disadvantageous arm as could be expected based on data of control rats in the current (vehicle infused IL rats) and past experiments (de Visser et al., 2011a; Koot et al., 2013). Therefore, we compared the data of the vehicle infused IL rats and vehicle infused lOFC rats (Table 1). Vehicle treated rats of the IL infused group decreased their fraction of visits to the disadvantageous arm, while vehicle treated rats of the lOFC infused group did not, which was confirmed by a repeated measures ANOVA (trial block: *F*_(2,46)_ = 4.389, *p* = 0.018; trial block ^*^ brain region: *F*_(2,46)_ = 3.280, *p* = 0.047; brain region: *F*_(1,23)_ = 4.916, *p* = 0.037). Remarkably, no differences were found for the empty arms (all *p* ≥ 0.401).

**Table 1:** **rIGT performance under treatment. Shown are means (± SEM) of fractions of visits to empty and disadvantageous arms of rats treated with vehicle in the infralimbic cortex (IL) or lateral orbitofrontal cortex (lOFC), for trial blocks 61-120**.

**rIGT performance (fraction of visits)**				
	**Empty arms**		**Disadvantageous arms**	
**Trial block**	**IL vehicle**	**IOFC vehicle**	**IL vehicle**	**IOFC vehicle**
61 − 80	0.33 ± 0.05	0.39 ± 0.05	0.40 ± 0.07	0.45 ± 0.07
81 − 100	0.29 ± 0.06	0.34 ± 0.06	0.29 ± 0.07	0.40 ± 0.07
101 − 120	0.29 ± 0.07	0.33 ± 0.07	0.14 ± 0.04	0.42 ± 0.04

## Discussion

Using a rodent version of the IGT to assess decision-making, this study resulted in two main findings. First, targeting the IL cortex, vehicle infused rats decreased the number of visits to the disadvantageous arm across trial blocks, while CORT infused rats did not. Infusions of CORT into the IL did not affect choices for empty arms. These data indicate that only reward-related decision-making was affected by CORT infusions. Second, targeting the lOFC cortex no differences were observed between vehicle and CORT treated rats in the number of visits to the disadvantageous or empty arms. However, compared to vehicle treated rats of the IL group, vehicle treated rats of the lOFC group showed no decrease in the number of visits to the disadvantageous arm. No differences were found for the empty arms, again suggesting that also here only reward-related decision-making was affected. The present results indicate that intracerebral infusions with CORT targeting the IL are sufficient to fully recapitulate the disrupted decision-making performance earlier observed with peripheral CORT administration.

### Methodological considerations

The selection of brain areas for infusion was based on a previous study (Koot et al., 2013): disrupted decision-making performance, as identified by a decrease of visits to the disadvantageous arm 30 min after systemic CORT injections, was related to significantly increased levels of c-Fos expression in the lOFC and insular cortex, and a trend in the IL. The current study aimed for elucidation of the effects of direct CORT administration into the IL and lOFC on decision-making in the rIGT. The insular cortex was left from this study due to technical difficulties targeting the insular cortex at the specific site that showed CORT-dependent changes in c-Fos expression after the rIGT.

The dose of CORT used for the infusions (5 ng/μL) was previously shown to enhance the effect on taste aversion memory when infused in the insular cortex or basolateral amygdala (Miranda et al., 2008) or on memory consolidation when infused in the dorsal striatum (Quirarte et al., 2009). Although in these studies other brain areas were targeted, the dose of 5 ng/μL in the present study was sufficient to induce significant differences between IL infused groups. As both vehicle and CORT treated rats of the OFC targeted groups showed a less than optimal decision-making performance, no conclusions on the dose for this brain region can be drawn (see further discussion below).

Previous studies with this variant of the rIGT have shown that male rats shift from exploratory behavior in the first half of the task to visiting the disadvantageous arms less often in the second half of the task (van den Bos et al., 2006, 2012; Homberg et al., 2008; de Visser et al., 2011a,b; van Hasselt et al., 2012). In the present study, we found that from trial 61 onwards vehicle treated rats within the IL infused group decreased their number of visits to the long-term disadvantageous arm. This suggests that cannulation and/or infusion procedure in the IL *per se* had no effect on rIGT performance. In contrast, cannulation and/or infusion procedure in the lOFC appeared to have an effect on rIGT performance as indicated by the poor decision-making performance (see below).

### Behavioral effects of CORT infusions targeting infralimbic cortex

Both the analysis of task-learning per group in relation to chance level and the analysis of performance differences between groups revealed the same finding: infusions of CORT into the IL affected choosing the advantageous arm over the disadvantageous arm within the baited arms, but had no effect on avoiding the empty arms. These data match the results that we found for systemic injections of CORT (Koot et al., 2013). In addition, following a significant interaction term only in the final trial block (101–120) a significant difference was found between CORT and vehicle infused rats in the baited arms. This interaction results from the fact that vehicle infused rats progressively decreased their choices for the disadvantageous arm while the performance of CORT treated rats remained at the same level. Only in the last trial block this difference in performance became sufficiently strong to reach significance. Again, these findings match the data that we obtained for systemic injections of CORT (Koot et al., 2013). Accordingly, while c-Fos analysis indicated a role for the IL in CORT mediated effects on rIGT decision-making, here we provide direct evidence by infusing CORT into the IL.

As both the IL and the overlying prelimbic cortex (PrL) play a role in rIGT task performance (de Visser et al., 2011a,b; Rivalan et al., 2011; van Hasselt et al., 2012; Paine et al., 2013), the present data could have been due to effects on the PrL. However, considering the injection angle (30°; avoiding thereby the PrL) and small volume (0.5 μL per hemisphere) injected at a slow rate (0.4 μL/min) in the current study, it is unlikely that the present CORT injections reached the PrL. Furthermore, the effect of inactivation of the PrL on rIGT performance has been shown to be dependent on whether rats still explored or were already showing a preference for the advantageous over the disadvantageous arm (de Visser et al., 2011b). This was not observed in the present study: the effects of CORT were independent on whether rats still explored or already showed a preference (data not shown). In addition, no effect on the PrL c-Fos activity was found following systemic CORT injections (Koot et al., 2013). Thus, the effects seem specific for the IL, suggesting that the IL is more sensitive to CORT related changes in decision-making than the PrL. Future experiments should elucidate this.

Corticosteroid actions in the brain are mediated by mineralocorticoid (MR) and glucocorticoid (GR) receptors (de Kloet et al., 2005) and the mPFC (IL and PrL) contains both MR and GR (Chao et al., 1989; Patel et al., 2000; Herman et al., 2005). After becoming activated by stress (Cullinan et al., 1995; Figueiredo et al., 2003) these receptors contribute to the neural control of the endocrine response and behavioral adaptation to stress (Diorio et al., 1993; Sullivan and Gratton, 2002; Herman et al., 2005). Shortly after stress nuclear-localised MR (nMR) levels are elevated in the mPFC, while nGR levels are not (Caudal et al., 2014). Only a few studies (Roozendaal et al., 2009; Barsegyan et al., 2010; Butts et al., 2011) delineated the direct effect of CORT applied within the mPFC (both PrL and IL), and found that impairments on working memory and improvements in memory consolidation were GR mediated. Additional experiments are needed to unravel whether the observed rapid effects of CORT infusion in the IL on decision-making are MR or GR mediated.

In an attempt to delineate the behavioral mechanisms underlying the significant difference between CORT and vehicle infused IL rats, the number of switches, win-stay and lose-shift behavior were examined, but no effects of CORT treatment were found on these parameters. This suggests that sensitivity to reward or punishment was not affected by the CORT infusions, as measured by a lack of effect on lose-shift or win-stay behavior. This is in line with our previous study (Koot et al., 2013), in which also working memory and memory consolidation did not seem to be affected. Furthermore, the selective effect on the baited arms supports this notion. These data show that CORT effects confined to the IL are sufficient to fully recapitulate the disrupted decision-making earlier observed after peripheral hormone administration.

The mPFC, including the IL, is critically involved in strategy shifting, behavioral flexibility and goal-directed learning behavior by encoding task-rules (Dias et al., 1996; Ragozzino et al., 1999; Birrell and Brown, 2000; Floresco et al., 2008; Tran-Tu-Yen et al., 2009; Young and Shapiro, 2009; Balleine and O’Doherty, 2010; Sul et al., 2010). Particularly, the IL promotes instrumental discrimination learning and suppresses impulsive responding (Chudasama and Robbins, 2003; Murphy et al., 2005, 2012). Inactivation of the IL leads to deficits in retrieval of emotional (extinction) memory (Sierra-Mercado et al., 2006). It has been suggested that the rodent IL constitutes a functional homologue of the human ventromedial PFC (Milad and Quirk, 2012). In line with this suggestion, patients with ventromedial PFC lesions also have difficulties in recalling the difference in emotional value between options prior to making the next choice within the IGT, hampering thereby task progression (Bechara et al., 1999). Thus, in the present study CORT infusion in the IL could have led to a disrupted IL functioning, leading to a lack of inhibitory control (Quirk and Beer, 2006). CORT infused rats may therefore continue to explore the different options in the rIGT rather than integrating and retrieving earlier information into a behavioral response, necessary for successful completion of the rIGT. The ventromedial PFC has been suggested to play a role in the IGT already in its early stages (Bechara et al., 1999; de Visser et al., 2011a). Thus, the possibility may be entertained to study the role of the IL in the early stages of the rIGT, i.e., in trials 1–60, to more fully capture its role in decision-making.

### Behavioral effects of CORT infusions targeting lOFC

In the lOFC infused group, no difference between vehicle and CORT treated rats in visits to the disadvantageous arm was found. Also no difference between vehicle and CORT treated rats in visits to the empty arms was observed. Therefore, we cannot draw conclusions as yet on the effect of CORT infusions targeting the lOFC. However, when comparing the two vehicle groups (IL and lOFC), vehicle treated lOFC cannulated rats did not decrease their visits to the disadvantageous arm, while vehicle treated IL cannulated rats did, just as other control rats in our previous studies (de Visser et al., 2011a; Koot et al., 2013). No differences were found in visits to the empty arms. This was also found when considering task-learning in vehicle treated lOFC rats in relation to chance level: while rats showed evidence of task-learning regarding the empty arms, no task-learning was observed regarding the disadvantageous arm. Based on these observations, it could well be that the lOFC was damaged in all rats of this group, as a result of the region’s cannulation, the volume infused on 3 consecutive days, or the insertion of longer injection needles prior to infusion. Nevertheless, we did not find any histological evidence for particular brain damage other than cannulation damage. Moreover, we cannot exclude that the lOFC (more so than the IL) was sensitive to the mild restraint necessary for the infusions and/or the 2% ethanol treatment in both the vehicle and CORT groups. It is difficult to define the effect of each of these procedures on the function of the lOFC, as to our knowledge no studies have been performed cannulating the lOFC for other purposes than inactivation or lesions of this area. Nonetheless, inactivation studies showed that the lOFC processes information about rewarding values (Kantak et al., 2009), and enables task switching by supporting initial inhibition of previously relevant choice patterns (Kim and Ragozzino, 2005; Ragozzino, 2007).

The disruption of reward-based decision-making in all lOFC cannulated rats in the present study fits within the frame of current knowledge of the role of the OFC in reinforcement-guided decision-making (Schoenbaum and Roesch, 2005; Fellows, 2007; Murray and Izquierdo, 2007; Pais-Vieira et al., 2007; Rivalan et al., 2011; Zeeb and Winstanley, 2011). The OFC is important in signaling the value of an expected outcome (Frank and Claus, 2006; Stalnaker et al., 2009), and damage to the OFC may prevent the adequate integration of information about the consequences of responding for a reward with the subjective value of that rewarding outcome (Schoenbaum et al., 1999; Ostlund and Balleine, 2007; Rivalan et al., 2011). Furthermore, the lOFC is important when a shift in choice pattern is demanded after a change in contingencies occurred and as such a suppression of previously rewarded behavior is required (Elliott et al., 2000; Bohn et al., 2003; Chudasama and Robbins, 2003; McAlonan and Brown, 2003; Fellows, 2007; Zald et al., 2014). Thus, our data suggest that the supposed lesions of the lOFC may have interfered with the ability to integrate knowledge of task contingencies into goal-directed behavior.

### Concluding remarks

The present study supports the crucial involvement of IL in the performance of male rats in the rIGT, as part of a network regulating decision-making. Similar to systemically injected CORT (Koot et al., 2013), CORT locally applied into the IL fully reproduced the disrupted reward-based decision-making performance of male rats in the rIGT earlier shown after peripheral administration. These findings complement previous studies indicating the role of prefrontal areas in decision-making and build on the existing knowledge of how corticosteroids can affect cognitive function. We were unable to delineate the effect of direct application of CORT on the lOFC in terms of behavioral outcome in the rIGT. Refined techniques, such as adjusted cannulation coordinates (less deep cannula, longer infusion needle) would be one option to circumvent possible lesions. Another option to resolve the incertitude could be continuation of testing (several) sessions after the last brain infusion.

Future studies should focus on whether CORT affects decision-making through mineralo- or GR receptors (Roozendaal et al., 2009; Barsegyan et al., 2010; Butts et al., 2011) and to what extent the results mimic the effects of acute stress. Additionally, in the future female rats should be included as corticosteroids in humans have a different effect on decision-making behavior in men than in women (van den Bos et al., 2009, 2014).

## Author contributions

Susanne Koot designed and performed experiments, analysed data and wrote the manuscript. Magdalini Koukou performed experiments and analysed data. Annemarie Baars, Peter Hesseling and José van ’t Klooster performed experiments. Marian Joëls and Ruud van den Bos supervised the project, designed the experiments and edited the manuscript.

## Conflict of interest statement

The authors declare that the research was conducted in the absence of any commercial or financial relationships that could be construed as a potential conflict of interest.
